# Preventable adverse drug events in critically ill HIV patients: Is the detection of potential drug-drug interactions a useful tool?

**DOI:** 10.6061/clinics/2018/e148

**Published:** 2018-02-12

**Authors:** Grazielle Viana Ramos, André Miguel Japiassú, Fernando Augusto Bozza, Lusiele Guaraldo

**Affiliations:** I Instituto Nacional de Infectologia Evandro Chagas, Fundacao Oswaldo Cruz, Rio de Janeiro, RJ, BR; II Instituto D’Or de Pesquisa e Ensino, Rio de Janeiro, RJ, BR

**Keywords:** Drug-Related Side Effects and Adverse Reactions, Drug Interactions, Intensive Care Units

## Abstract

**OBJECTIVES:**

The aim of this study was to develop a strategy to identify adverse drug events associated with drug-drug interactions by analyzing the prescriptions of critically ill patients.

**METHODS:**

This retrospective study included HIV/AIDS patients who were admitted to an intensive care unit between November 2006 and September 2008. Data were collected in two stages. In the first stage, three prescriptions administered throughout the entire duration of these patients’ hospitalization were reviewed, with the Micromedex database used to search for potential drug-drug interactions. In the second stage, a search for adverse drug events in all available medical, nursing and laboratory records was performed. The probability that a drug-drug interaction caused each adverse drug events was assessed using the Naranjo algorithm.

**RESULTS:**

A total of 186 drug prescriptions of 62 HIV/AIDS patients were analyzed. There were 331 potential drug-drug interactions, and 9% of these potential interactions resulted in adverse drug events in 16 patients; these adverse drug events included treatment failure (16.7%) and adverse reactions (83.3%). Most of the adverse drug reactions were classified as possible based on the Naranjo algorithm.

**CONCLUSIONS:**

The approach used in this study allowed for the detection of adverse drug events related to 9% of the potential drug-drug interactions that were identified; these adverse drug events affected 26% of the study population. With the monitoring of adverse drug events based on prescriptions, a combination of the evaluation of potential drug-drug interactions by clinical pharmacy services and the monitoring of critically ill patients is an effective strategy that can be used as a complementary tool for safety assessments and the prevention of adverse drug events.

## INTRODUCTION

Critically ill patients are at high risk of adverse drug events (ADEs) for several reasons, including the complexity of their clinical conditions, which can involve pharmacokinetic variations and concurrent treatment with multiple drugs 1. These events can seriously affect patients’ evolution and frequently complicate clinical management by increasing lengths of hospital stay and medical costs 2,3.

An ADE has been defined as “any injury occurring during the patient’s drug therapy and resulting either from appropriate care or from unsuitable or suboptimal care” and includes adverse drug reactions (ADRs) during the recommended use of a medication and any harm secondary to a medication error 4.

There are several methods for detecting ADE. Spontaneous reporting is the traditional approach for ADE detection and is often used in systematic analyses of ADEs 5. However, such monitoring has the disadvantage of being more vulnerable to underreporting and lack of detail; this drawback hampers analysis 6. Another method for ADE detection is active surveillance based on focused and structured activity that includes direct observation, patient chart audits and trigger tools. This strategy is often used in pharmacovigilance studies in the ICU 7.

A large proportion of ADRs are known and preventable, and such reactions are often attributable to the coadministration of drugs with an established interaction 6. Recent publications have shown that many potential drug-drug interactions (DDIs) can be identified using drug prescriptions 2,8.

HIV patients admitted to the ICU typically have multiple comorbidities and require frequent and concurrent therapeutic schemes in combination with antiretroviral drugs; as a result, DDI risks are elevated for such patients 9. In a previous study involving the use of two electronic databases, we detected a high number of potential DDIs associated with the prescriptions of critically ill HIV patients 10.

Although literature reports have revealed high estimates of potential DDIs in critically ill patients, few studies have correlated DDIs with clinically relevant ADEs. The aim of this study was to evaluate a strategy to identify ADEs using potential DDIs identified from the prescriptions of critically ill HIV patients.

## METHODS

This retrospective study included all adult HIV/AIDS patients (≥18 years of age) who were admitted to the ICU at Evandro Chagas National Institute of Infectious Diseases (INI) between November 2006 and September 2008 and required mechanical ventilation. Patients participating in clinical trials or patients with a length of ICU stay of less than 72 hours were excluded. This cohort was used because a high number of contraindicated drug combinations and major potential DDIs in this population had been identified in a prior study 10.

We collected information about prescribed drugs, length of ICU stay, ADEs and demographic/clinical data from both medical records and the INI ICU’s database. Simplified Acute Physiology Scores (SAPS II) were calculated to assess the severity of acute illness 11.

Data were collected in two consecutive stages. In the first stage, three prescriptions for each patient were analyzed at the following time points: 1) 24 hours after admission to the ICU; 2) the median length of hospitalization; and 3) the patient’s ICU discharge or death. The administered drugs were recorded at these time points, and potential DDIs were evaluated using the Micromedex 2.0 12 database. This database can help identify DDIs and provides information about their clinical consequences. In addition, in accordance with Micromedex definitions, DDIs were classified based on their mechanism of action (pharmacokinetic or pharmacodynamic), severity (contraindicated, severe, moderate, mild or unknown), level of evidence based on the quality of documentation (excellent, good, fair, poor, unlikely or unknown) and time of onset (immediate or delayed).

For the purposes of this analysis, given the clinical relevance of potential DDIs, only moderate, severe and contraindicated interactions with a level of evidence classified as either excellent or good were examined. Drug interactions present in sedation protocols or in treatments for tuberculosis were not considered in this analysis.

Potential interactions and other variables were recorded in a standardized and tested form. Drugs involved in potential DDIs were categorized according to their Anatomical Therapeutic Chemical classification (ATC/DDD 2010) 13.

In the second stage of this study, a search for ADEs in patients’ medical records was performed. Based on potential DDIs identified in the prior stage and their clinical consequences, we examined medical records to detect ADEs. Data were obtained from medical and nursing notes and from laboratory test results provided in patients’ medical charts.

In addition, the probabilities that the identified ADEs were caused by potential DDIs were assessed using the Naranjo algorithm, a ten-item scored questionnaire used to classify such events as “definite”, “probable”, “possible” or “doubtful”. In our study, to facilitate appropriate classification, we considered each pair of drugs involved in an interaction, comorbidities and other medications 14.

Data were entered into EpiData 3.1 and analyzed using SPSS for Windows, version 16.0. Exploratory analyses of demographic data, DDIs and ADEs were performed using frequencies, medians and ranges.

### Ethical Approval

The authors confirm their adherence to ethical principles during all phases of this study. This study was approved by the Institutional Review Board of INI.

## RESULTS

We analyzed the medical records of 62 HIV/AIDS patients who required mechanical ventilation. These patients had a median age of 37.5 years and were predominantly male (72.6%). The median SAPS II was 56 points (range, 31-91 points), and the length of stay in the ICU ranged from 3 to 122 days, with a median of 13 days. A total of 186 prescriptions were analyzed, with a mean of 9 drugs per prescription.

**Figure 1:**
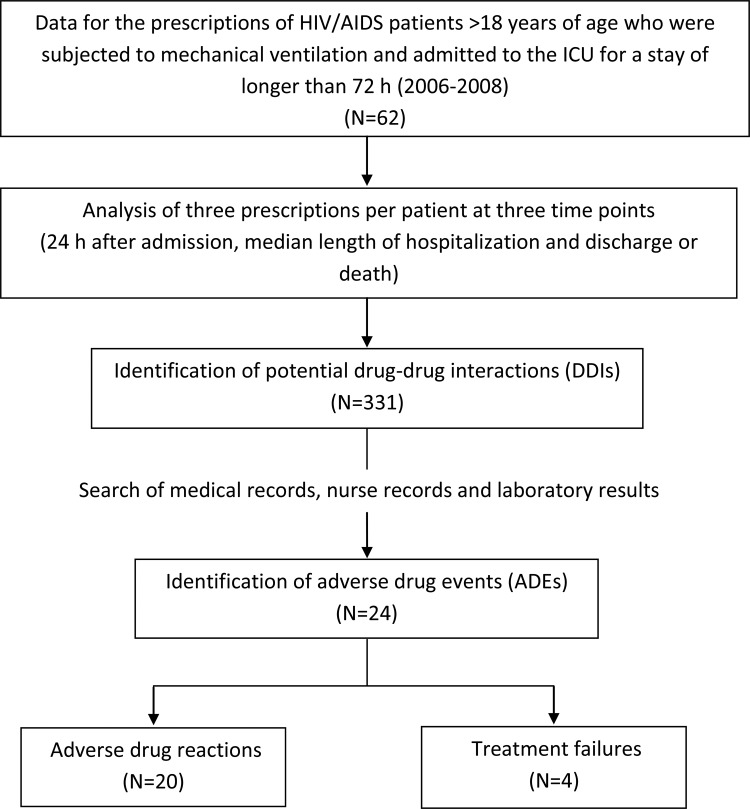
Flowchart of the study.

For all analyzed prescriptions, 331 potential DDIs were identified using the Micromedex database (Figure 1). The predominant characteristics of these DDIs were moderate interaction (74.0%), a delayed onset (63.7%), a pharmacokinetic mechanism (68.3%) and good scientific documentation (65.9%). We found that 9% of the identified DDIs were related to ADEs; 24 ADEs were identified in 16 patients (Table 1). ADRs accounted for 83.3% of the identified ADEs and were mostly classified as moderate and possible. There were four cases (16.7%) of therapeutic failure; the most common ADE was seizure during anticonvulsant treatment.

The combination of fluconazole and omeprazole was the most frequent pair of drugs involved in DDIs and associated with ADEs. In particular, this combination was thought to be involved in 6 of the 24 ADEs; these ADEs were related to elevated transaminase levels and diarrhea.

## DISCUSSION

The results suggest that our approach for identifying ADEs using potential DDIs is feasible. It was possible to identify a high frequency of potential DDIs from prescriptions; 9% of these potential DDIs resulted in ADEs and affected 26% of the study population.

A higher percentage of patients with ADEs was observed in this study (26%) than in prior investigations. Krahenbul-Melcher et al. 15 reported a corresponding percentage of less than 5%, whereas Reis and Cassiani 8 observed ADE-related potential DDIs in 7% of critically ill patients. The elevated frequency observed in our study is likely due to the examination of a cohort of critically ill HIV patients. The treatment of such patients with combinations of multiple drugs and antiretrovirals presents the potential for DDIs, an important cause of ADRs 16. The fact that four ADEs associated with five clinically relevant DDIs involving antiretroviral drugs were identified in our study may demonstrate the importance of the evaluation of therapeutic management in these patients.

The percentage of therapeutic failures observed in our study is similar to that observed by Reis and Cassiani 8, who found that 17.5 to 19% of potential DDIs detected based on the prescriptions of critically ill patients at a university hospital were related to therapeutic failures.

The probability of ADRs caused by a DDI was assessed using the Naranjo algorithm. We observed that most of the observed ADRs were classified as possible (80%); this result was similar to that obtained by Bucşa 17, who reported that 71.4% of ADRs related to DDIs were classified as possible.

The Naranjo algorithm has proven to be useful for both prospective and retrospective diagnoses of ADRs in ICU patients 1,2. However, given the categories of causality associated with this algorithm, many studies regard only events categorized as having a probability greater than possible as ADRs 2. From this perspective, these results point to the effectiveness of an ADR tracking method involving the consideration of potential DDIs.

Given the complexity of the analyzed population and the methodology that we used, our results should be evaluated carefully. We analyzed a specific subgroup of critically ill (HIV-infected) patients, and our results may not be applicable to other patients. Furthermore, retrospective data collection based on chart review can generate bias due to incomplete patient records. To minimize this potential bias, we used medical and nursing notes as well as laboratory results to identify ADEs. Another limitation is the small cohort included in this study, although we sought to minimize this limitation by analyzing 3 prescriptions per patient, resulting in 186 days of observation.

Despite these limitations, the presented approach provides improvements with respect to safety in pharmacotherapy for critically ill patients in intensive care. This method also contributes to improving knowledge of the occurrence of DDIs and ADEs and their impacts on the outcomes of critically ill patients by accounting for clinical complexity, polypharmacy and other factors that hamper the detection and evaluation of ADEs in these patients.

In conclusion, this method could be used to detect ADEs, including therapeutic failures and ADRs; such events were related to 9% of identified DDIs. Given the complexity of ADE evaluation in ICU patients, the strategy of monitoring ADEs by examining potential DDIs in prescriptions can be effective for identifying and preventing relevant events in critically ill patients, and this approach should be used as a complementary tool in safety assessments.

## AUTHOR CONTRIBUTIONS

All authors substantially contributed to the study design and methods. Ramos GV, Guaraldo L, Japiassú AM and Bozza FA conceived the study. Ramos GV and Japiassú AM collected clinical data. All authors performed data analysis. Ramos GV, Guaraldo L and Bozza FB drafted the manuscript, and Japiassú AM critically revised it for important intellectual content. All authors read and approved the final version of the manuscript.

## Figures and Tables

**Table 1:** Adverse drug events related to drug-drug interactions in patients admitted to the ICU at INI (N=24).

Adverse drug event		Drug-drug interaction	N
Treatment failure	Seizure	Valproic acid×ritonavir	3
		Phenytoin×rifampin	
		Phenytoin×ritonavir	
		Acyclovir×phenytoin	
		Ciprofloxacin×phenytoin	
	Poor sedation	Morphine×rifampin	1
		Fentanyl×rifampin	
Adverse drug reaction	Elevated transaminase levels	Fluconazole×omeprazole	3
		Fluconazole×prednisone	
	Diarrhea	Fluconazole×omeprazole	3
	Excessive sedation	Fluconazole×midazolam	3
		Clonazepam×ritonavir	
	Oral bleeding*	Pyrimethamine×SMX/TMP**	2
	Hypotension	Amlodipine×fluconazole	1
	Depression	Prednisone×ritonavir	1
	Hepatotoxicity	Phenytoin×acetaminophen	1
	Hypertension	Clarithromycin×prednisone	1
	Pancytopenia*	Pyrimethamine×SMX/TMP**	1
	Cardiac arrest*	Amiodarone×fentanyl	1
		Amiodarone×rifampin	
		Clarithromycin×fluconazole	
	Somnolence	Risperidone×ritonavir	1
	Vomiting	Diazepam×phenytoin	1
	Anxiety	Clarithromycin×prednisone	1
		**Total**	**24**

* serious interactions

** sulfamethoxazole/trimethoprim
